# Progress of Exosomal LncRNAs in Pancreatic Cancer

**DOI:** 10.3390/ijms25168665

**Published:** 2024-08-08

**Authors:** Chengyan Wei, Chunwei Zhang, Yuanzhi Zhou, Jingjing Wang, Yong Jin

**Affiliations:** School of Pharmacy, Anhui Medical University, Hefei 230032, China

**Keywords:** exosomes, LncRNA, exosomal LncRNAs, pancreatic cance, functions

## Abstract

Pancreatic cancer is a prevalent malignant tumor with rising medication resistance and mortality. Due to a dearth of specific and trustworthy biomarkers and therapeutic targets, pancreatic cancer early detection and treatment are still not at their best. Exosomal LncRNAs have been found to be plentiful and persistent within exosomes, and they are capable of functioning whether the exosomes are traveling to close or distant cells. Furthermore, increasing evidence suggests that exosomal LncRNA, identified as an oncogene or tumor suppressor-control the growth, metastasis, and susceptibility of pancreatic cancer to chemotherapy and radiation therapy. Promising prospects for both antitumor targets and diagnostic biomarkers are exosomal LncRNAs. The primary features of exosomal LncRNAs, their biological roles in the onset and progression of pancreatic cancer, and their potential as therapeutic targets and diagnostic molecular markers are outlined in this review.

## 1. Introduction

Pancreatic ductal adenocarcinoma and pancreatic endocrine tumors are the two primary pathological kinds of pancreatic cancers (PACA) [1]. The glandular epithelium’s pancreatic ductal adenocarcinomas account for 85% of PACA. They are among the digestive system’s malignant tumors that are difficult to identify early, have an elevated rate of mortality [2] and a dismal prognosis [3], and are among the leading causes of cancer-related deaths globally [2,4,5]. They are also the fourth most prevalent cause of cancer-related deaths. Pancreatic cancer progresses quickly; by the time a patient is diagnosed, most have either reached an advanced stage or have even metastasized. Patients with pancreatic cancer often exhibit nonspecific symptoms, such as weight loss and abdominal pain [5,6], which can make diagnosis challenging. More than half of the cases were only found in phase IV, only 20% of cases can be removed when diagnosed, and unfortunately the 5-year survival is only 5–10%, with a median survival time of 5–6 months after diagnosis [7,8]. According to statistics, PACA incidence and mortality are rising annually in some countries (such as the US, China, and Europe), which may be connected to the average age of most populations rising. Additionally, men are more likely than women to experience PACA incidence and mortality [9,10]. Age affects the likelihood of getting PACA; those over 70 have the highest risk, and 90% of patients receive a diagnosis after the age of 55 [11,12]. Apart from surgery, chemotherapy is the primary adjuvant for pancreatic cancer; nevertheless, there are still few treatments available, such as palliative therapy, targeted therapies, stroma targeting and immunotherapies. With a 5-year survival rate of roughly 31.5%, it is anticipated that only 9% of PACA patients are in the stage of localized disease and can be treated with surgical resection and radiation therapy to gain a better prognosis [13,14]. Currently, the most common and successful surgical treatment for treating early-stage PACA is pancreaticoduodenectomy; patients with intermediate- to late-stage PACA are primarily treated with radiotherapy [15]. However, current radiation treatments are ineffective and prone to medication resistance, which worsens prognoses because of the heterogeneity of PACA patients and the unclear etiology. The traditional tumor marker CA19-9 is used to diagnose pancreatic cancer; however, because of its low diagnostic sensitivity and specificity, it is also not very useful in the early identification of PACA [16]. The first-line chemotherapies for patients with advanced pancreatic cancer include 5-fluorouracil/folinic acid with irinotecan and oxaliplatin (FOLFIRINOX) and gemcitabine plus nanoparticle albumin-bound paclitaxel (nab-paclitaxel) [17,18,19,20,21,22]. For patients, surgical excision is believed to be the sole drastic treatment option [23]. Regrettably, chemoresistance typically lowers the efficiency of chemotherapy drugs, worsening the prognosis for patients with metastatic cancer. Surgical excision also typically causes a temporary relapse [24,25,26,27]. Early detection, halting metastases, and looking into effective therapy options are critical to the management of pancreatic cancer. In order to prolong patient life span, improve prognoses, and deliver efficient PACA treatment, it is crucial to research into the molecular mechanisms behind PACA development and search for biomarkers for early identification.

Aberrant glycosylation is characteristic of most cancers [28]. Glycosylation regulates key steps in cancer biology, including tumor invasion, metastasis, and cell signaling [29,30,31,32]. In addition, abnormal glycosylation is an important indicator of induction of tumor-free regulation because it provides T cells with a recognition antigen [28,33]. Abnormal glycosylation patterns found in pancreatic adenocarcinoma include sialylation, abnormal branching, O-glycan structure, fucosylation, and mucin alterations [34]. This alters multiple tumor-promoting signaling pathways, enhances metastatic phenotypes, and reshapes the tumor immune microenvironment [35]. However, glycosylated molecules have not been extensively studied as prognostic markers in pancreatic cancer. The aim of this study was to identify glycosylation-associated genes in pancreatic cancer and use them to construct reliable prognostic models.

The study had some limitations. First, it uses public datasets without using in vitro or in vivo models to validate or validate the results. Therefore, glycosylation-associated features must be validated in large-scale prospective studies to demonstrate their robustness. However, there is no gold standard approach in the evolving field of GRGs and their role in cancer. As a result, as many analytical strategies and sources as possible were integrated.

In conclusion, the results of this study provide insights into the role of glycosylation-related genes in predicting clinical outcomes in pancreatic cancer.

## 2. Exosomes

### 2.1. Characteristics of Exosomes

**Figure 1:**
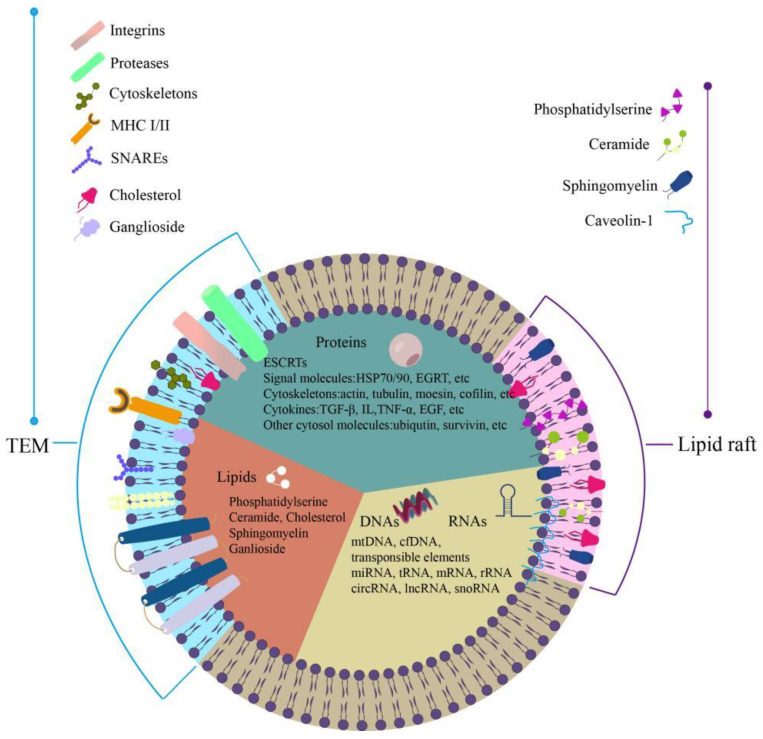
Exosomes mainly consist of spherical, disc, or cup-shaped nanoparticles, coated by phospholipid bilayer, with a diameter of 40–150 nm, these nanostructures typically contain proteins, nucleic acids, lipid molecules, and other inorganic substances. Exosomes can be generated by various types of cells, they all share similar structural proteins, such as major histocompatibility complex class I and class II molecules (MHC I/II).

The majority of exosomes are composed of spherical, disc- or cup-shaped nanoparticles with a diameter of 40–150 nm (Figure 1) that are coated in a phospholipid bilayer [36]. These nanostructures often contain proteins, nucleic acids, lipid molecules, and other inorganic elements Ca^2+^. While various cell types are able to produce exosomes, all of these cell types have structural proteins in common, including Rab GTPases, major histocompatibility complex (MHC I and II), annexins, tumor susceptibility gene 101 protein (TSG101), flotillin (FLOT1), integrins, and tetraspanins (Tspans) [37].

The four transmembrane proteins that make up tspans are mostly CD9, CD63, CD81, CD82, and so on. These proteins are approximately 100 times more abundant in exosomes than they are in their parent cells. The formation of heterocomplexes or homodimers between Tspans and other proteins is possible. Additionally, Tspans can pair with gangliosides and cholesterol to form unique Tspan-enriched microdomains (TEMs). TEMs can function as a particular signal transduction platform by selectively recruiting membrane-associated proteins like integrins, proteases, and other related signal molecules, contingent on the demands of the cell [38].

### 2.2. Exosome Secretion and Uptake

**Figure 2:**
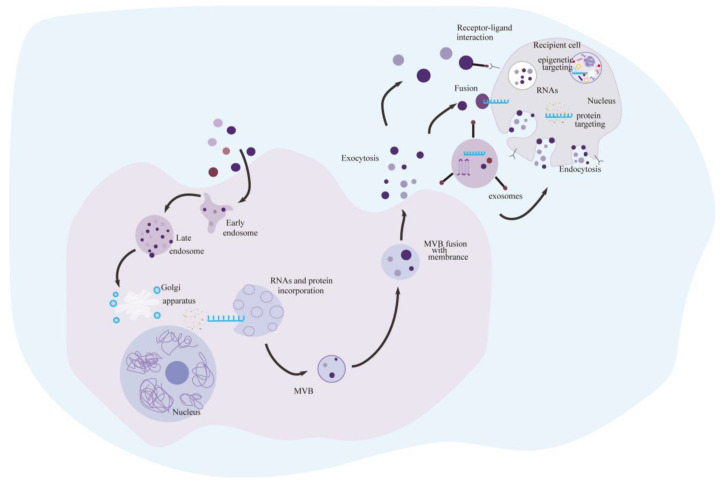
The biogenesis and contents of exosomes. Early nuclear endosomes are formed when the plasma membrane buddings inward and merges with membrane proteins. Nuclear endosome invagination and the encapsulation of certain materials, including proteins and nucleic acids, Following their fusion with the plasma membrane, these multivesicular bodies (MVBs) release exosomes into extracellular locations. Exosomes facilitate interactions between donor and recipient cells through multiple pathways, including direct fusion; receptor-ligand interactions, and endocytosis.

Originally, extracellular vesicles (MVBs) with nucleotidase activity that are released by a range of different cells and measure between 30 and 100 nm in diameter were referred to as exosomes (Figure 2) [39]. Later, an exosome was identified as a double layer of extracellular vesicles secreted by several cell types, especially malignant cells. These nanoscale vesicles are produced within multivesicular endosomes and are formed by fusing with the plasma membrane via MVB [40]. However microvesicles and exosomes are thought to germinate directly from the plasma membrane. The inward germination of the plasma membrane to create early endosomes is the first step in the production of exosomes [41]. Endosomes bud inward in a restricted area to generate nanoscale vesicles during endosome maturation, resulting in MVBs containing intraluminal vesicles (ILVs) [42,43], which contain cytoplasmic components, such as soluble proteins and nucleic acids. ILVs merge with the plasma membrane through MVBs during the formation of MVBs [44].

By directly connecting transmembrane proteins or lipid ligands with cell surface receptors, secreted exosomes contribute to basic pleiotropic biological processes. Membrane fusion is then used to transfer cytoplasmic proteins and nucleic acids to the recipient cells. This characteristic may enable them to function as letter carriers in cellular contacts and to be important players in the development and advancement of several diseases, such as tumor growth, metastasis, and immunological escape. Numerous normal, malignant, and immune cell types can release exosomes [45,46]. Compared to normal cells, tumor cells release more exosomes [47]. Cells in close proximity or far away can absorb the released exosomes [47]. Research into exosomes is very popular right now. Notably, tumor-derived exosomes play a critical role in the growth and dissemination of malignancies. Numerous studies have shown that exosomes are significant mediators of intercellular communication between stromal cells and tumor cells in both proximal and distant microenvironments [48]. Exosomes can be isolated from a wide range of biological fluids, such as blood [49], urine [50], saliva [51], and breast milk [52]. Exosomes are mostly composed of proteins, lipids, and nucleic acids. While all exosomes contain all other proteins and lipids, some exosomes are enriched in specific proteins, lipids, and nucleic acids [53,54,55]. Additionally, endosomal-specific tetraproteins and MVB biogenesis-associated proteins are present on the membrane surface of exosomes [56]. A noninvasive biomarker for a range of cancers may one day be employed to assess exosome viability in body fluids.

Exosomes are now understood to be significant information-flow carriers. Since these biological components are transferred via exosomes, they have a major role in the progression of cancer. The increasing interest in exosomes as drug delivery methods for cancer or nucleic acid therapy arises from their stability, specificity, and accessibility. Furthermore, exosomes have negligible harmful effects and are nonimmunogenic [57]. Thus, there is a lot of promise for the practical application of exosomes containing therapeutic biological molecules in cancer therapy.

## 3. Exosomal LncRNA

The two main types of RNA are coding RNA and noncoding RNA. Based on their size, noncoding RNAs are further separated into small ncRNAs and long ncRNAs (LncRNAs). Among the numerous RNAs found in large intergenic noncoding RNAs (LncRNAs) are cyclic RNAs (circRNAs) and miRNAs. One of the most lethal cancers involving the digestive tract and linked to the dysregulation of miR-129-5p, is pancreatic cancer [58]; tiny ncRNAs, on the other hand, are less than 200 nucleotides and mostly consist of small interfering RNAs and miRNAs. LncRNAs are noncoding RNAs longer than 200 nucleotides that have little to no ability to code for proteins [59]. LncRNAs have interactions with DNA, RNA, and proteins. They are found in a cell’s nucleus or cytoplasm [60]. LncRNAs have significance. Long noncoding RNAs (LncRNAs) have been shown to play important roles in a variety of physiological processes, including cell division, apoptosis, migration, and senescence [61,62]. Expanding studies have demonstrated that LncRNAs impact gene expression, either directly or indirectly, and that this is connected to the development and metastasis of cancer [63,64,65]. LncRNAs can be selectively sorted into exosomes and act as signaling messengers in intercellular communication [66]. Exosomal LncRNAs have the ability to go to recipient cells and impart phenotypic changes. LncRNAs derived from exosomes have been found to be involved in tumor growth, angiogenesis, metastasis, and resistance to treatment [67]. Furthermore, exosomal LncRNAs have the ability to rewire cells inside the tumor microenvironment, which facilitates the growth of tumors. LncRNAs can be released into a variety of bodily fluids after being encapsulated in exosomes [68,69]. Body fluids are stabilized and LncRNAs are shielded from ribonuclease-mediated destruction by exosomes [70]. It is possible that exosomal LncRNAs could be used as biomarkers for different kinds of cancer [71,72]. As a result, LncRNAs are getting more and more attention in the exosome research community.

## 4. Biological Functions of Exosomal LncRNAs in PACA

Usually, in transmission electron microscopy (TEM) images, exosomes are translucent, cup-shaped, or spherical structures. Exosomes have been reported as carriers of miR-NAs, LncRNAs, proteins, and even circRNAs for intercellular signaling [73,74,75]. Research has demonstrated that the body’s outermost LncRNAs are substantially enriched. This could be because the body secretes protective qualities or a certain protein structure sequence and/or its variant. Due to the bilayer membrane of the exosome, the payload is protected from clearance or degradation. The results show that the abundance of LncRNAs provides a novel and valuable resource for studying the physiology and pathology of the body. Therefore, LncRNAs have attracted more and more attention in the study of exosomes.

**Figure 3:**
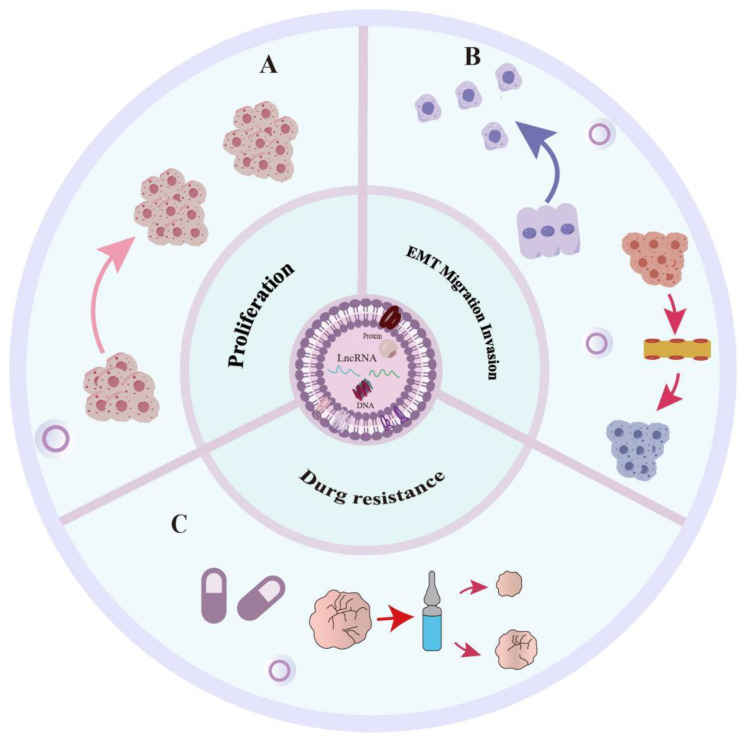
Mechanisms of exosomal LncRNA-mediated action in PACA. (**A**) Exosomal lncRNA affects the proliferation of pancreatic cancer. (**B**) Exosomal lncRNA affects the Epithelial-Mesenchymal Transition (EMT), migration, and invasion of pancreatic cancer. (**C**) Exosomal lncRNA affects the drug resistance of pancreatic cancer.

An increasing number of studies have demonstrated a close correlation between the release of several exosomal LncRNAs and the onset and development of pancreatic cancer. Exosomal LncRNAs, once ingested by recipient cells, govern a range of functions, including drug resistance, invasion, migration, and proliferation of tumors. (Figure 3 and Table 1).

### 4.1. Proliferation

One of the fundamental features of human cancers is the overgrowth of malignant cells. Typically, genes associated with cancer regulate cell growth by means of proliferation. Exosomal LncRNAs are involved in this process.

Upregulation of LncRNAUCA1 in PDAC cells promotes cell proliferation, migration and invasion [76]. Mechanistic research has demonstrated that the AKT and ERK signaling pathways are activated [76]. Moreover, UCA1 directly targets miR-135a to promote the growth and dissemination of pancreatic cancer cells [84]. Hippo signaling pathway [85] and miR-96/FOXO3 axis are also implicated in UCA1-mediated enhancement of PDAC development. Additionally, UCA1 overexpression in pancreatic cancer enhances PDAC cells’ stemness and cell proliferation by splashing miR-590-3p and interacting with hnRNPA2B1 to increase oncogenic KRAS expression and activation [86]. Thus, LncRNA UCA1 is considered a promising target for stopping PDAC progression and drug susceptibility, and it is worth more research.

Current studies show that the serum of PC patients has significant levels of expression of LncRNA SNHG11 (SNHG11). Exosome-mediated SNHG11 can be downregulated by miR-11-324p or upregulated by VEGFA, which can reverse the inhibitory effects of SNHG3 depletion on cell proliferation, migration, and angiogenesis. Additionally, transfection with pcDNA3.1-VEGFA or miR-324-3p inhibitor can rescue the proapoptotic effects of SNHG11 silencing on apoptosis, potentially leading to the creation of novel PC biomarkers [77].

There is proof that M2 macrophage exosomes’ expression of the LncRNA SBF2-AS1 influences PANC-1 cells’ capacity to proliferate tumorigenesis in nude mice, and M2 macrophage exosomes promote the development of PACA cells. SBF1-AS2 LncRNA restriction in exosomes produced from M2 macrophages helped to inhibit PC cells’ capacity to spread tumors. It was found that LncRNA SBF2-AS1 inhibits miR-122-5p and upregulates XIAP as a competitive internal RNA [87].

### 4.2. Migration, Invasion, EMT

One significant factor raising the risk of cancer death is metastasis [88,89]. Invasiveness and metastasis are the primary causes of therapy failure in pancreatic cancer. Cancer cells can travel and invade far-off places to enter. Exosomes regulate PC invasion and metastasis through their regulatory effects on pancreatic cancer cells and the tumor microenvironment. It is thought that exosomes contribute to the proliferation of cancer cells. Several studies have shown the roles that exosomes derived from tumors play in invasion and metastasis [90,91,92].

PACA tissues and cells exhibited elevated levels of LINC00460, and its knockdown promoted apoptosis and G0/G1 phase block while also slowing down cell growth, migration, and invasion. Tumor development was also inhibited by anti-PD-1 treatment [93]. ANLN upregulation and miR-503-5p downregulation counteracted the effects of LINC00460 knockdown on the inhibition of proliferation, migration, and invasion in addition to the promotion of apoptosis. Exosomes generated from panc-1 cells accelerated cell invasion and migration by polarizing M2 macrophages, a response induced by LINC00460. As a result, LINC00460 may be an option for PC medical treatment [78].

Chen and colleagues discovered that exosomes produced from PDAC expressed 3227 LncRNAs. Subsequent investigation demonstrated that LncRNA ENST00000560647 was significantly expressed in exosomes and interacted with miRNAs linked to pancreatic cancer. Furthermore, dendritic cells were treated with PDA-derived exosomes in order to comprehend LncRNA expression. When compared to control dendritic cells (DCS), the expression of LncRNAENST00000560647 rose sevenfold following treatment with exosomes. It is likely that the exosomal transfer of ENST00000560647 contributes significantly to immunological escape from PDAC; however, more research is required to identify the factors that may influence their expression in PDAC [79].

Exosomal LncRNAs play a role in PACA cell metastasis and stemness. By acting as ceRNAs to lower the expression level of miRNA-200, exosomes harboring the LncRNA Sox2ot can cause EMT and PACA cell metastasis. Sox2ot also increases SOX2 expression to improve PACA cell stemness [80].

Significant metabolic stress, typically caused by glucose and glutamine depletion, is also associated with the development of pancreatic cancer. This metabolic stress results in the overexpression of ZNFX1 antisense RNA1 (ZFAS1), which is crucial in promoting PACA cell metastasis and the epithelial-mesenchymal transition (EMT). ZFAS1 is a potential novel therapeutic target for PACA metabolic therapy and mechanistically improves the relationship between the primary regulator of EMT, ZEB1, and the important kinase AMPK [81].

Additionally, Wnt/β-linker protein promotes EMT to increase PACA cell migration and metastasis. To increase PACA cell migration and induce EMT, osteopontin secretes LINC01133-rich exosomes. LINC01133 promotes EMT-mediated PACA metastasis by activating Wnt/β-linker protein signaling and reducing AXIN2 expression through EZH2 recruitment [82].

### 4.3. Drug Resistance

Chemotherapy resistance remains one of the main problems with cancer treatment [94,95]. Gemcitabine has been a widely utilized chemotherapeutic drug in recent years, and it is the first-line treatment for advanced pancreatic cancer [96,97,98]. Even while medications like gemcitabine help patients with pancreatic cancer, the effectiveness of chemotherapy is significantly limited by the development of resistance to gemcitabine. It has been established that hypoxia promotes tumor growth and treatment resistance by inducing exosome secretion. Second, it was discovered that tumor cells might be transfected by exosomes released by pancreatic cancer cells in either normoxic or hypoxic environments. By transferring exosomal long noncoding RNA reprogramming regulator (lncROR), hexo inhibited the Hippo/Yes-associated protein (Hippo/YAP) pathway in pancreatic cancer cells, promoting resistance to gemcitabine and preventing gemcitabine-induced apoptosis and cell cycle arrest. Consequently, exosomal lncROR may be a viable target for chemotherapy in pancreatic cancer [83].

Exosomal LncRNAs have been demonstrated in these studies to impact a number of target cell molecular pathways, including Wnt signaling, which in turn affects PACA cell invasion and proliferation. Future research should address these two unanswered questions: (i) how exosomal LncRNAs affect PACA cells’ treatment response; and (ii) how exosomal LncRNAs function as prognostic and diagnostic indicators in PACA.

## 5. Exosomal LncRNA as a Potential Biomarker for PACA

Exosomal LncRNA expression and composition changes typically correspond to alterations in the clinical state of cells. Given their involvement in the development and genesis of cancer, exosomal LncRNAs may be useful as therapeutic targets and biomarkers for the detection, assessment, and treatment of cancer (Table 2) [99,100]. PACA has significant death rates because it has few distinct symptoms in the early stages and is frequently detected in the latter stages [101]. Conventional imaging studies are frequently employed in the clinical assessment of PACA, including CT, MRI, and ultrasound. Despite being the sole FDA-approved biomarker for PACA, CA19-9’s accuracy is far from ideal because of its low overall specificity and poor early sensitivity. The only biomarker for PACA that has received FDA approval is CA19-9, but its accuracy is far from ideal because of its low overall specificity and poor early sensitivity [102,103,104]. Thus, there is an immediate need for a noninvasive diagnostic technique that is more accurate. Since exosomes contain a variety of biological payloads, as was previously discussed, they have drawn attention to possible biomarkers for the early detection of certain cancers [105]. Exosomal LncRNA is one of them that is becoming known as a new biomarker for PACA [106,107,108,109,110].

There is proof that peripheral blood contains EV (extracellular vesicle)-associated LncRNAs with increased SOX2OT expression, with volumes that match tumor sizes [80]. Additionally, compared to circulating tumor markers frequently utilized in the field, serum EV findings used as tumor biomarkers have demonstrated promising diagnostic accuracy utilizing RNA sequencing data [71,111]. Another recent study focusing on HULC, which can be metastasized by EV and drives EMT, invasion, and migration in recipient PDAC cells, offers similar benefits in terms of diagnostic and prognostic indicators for PDAC [112,113]. PDAC in humans may be detected in the bloodstream by extracellular vesicle-encapsulated human lung cancer (HULC) [114]. Takahashi et al. examined and contrasted circulating HULC with CA19-9 in individuals with PDAC. Similar to CA19-9, HULC was utilized to distinguish between PDAC and healthy patients, with an AUC value of 0.92 [114]. The findings demonstrated that the HULC biomarker’s diagnostic capacity was more effective in distinguishing between PDAC patients and healthy individuals, and that patients with pancreatic cancer and those with benign pancreatic diseases could be distinguished from one another using the biomarker’s high expression of HULC in serum [115]. Additionally, it has been demonstrated that HULC is a useful serum biomarker for both the diagnosis and prognosis of stomach cancer [116].

Interestingly, a recent study looked at the RNA cargo of exosomes in the blood of healthy people, intraductal papillary mucinous neoplasia (IPMN), and PDAC patients [117]. They discovered that, in contrast to healthy exosomes, IPMN and PDAC serum exosomes specifically overexpressed the LncRNA MALAT1 and showed colorectal tumor differential expression (CRNDE) [117,118].

**Table 2:** Exosome RNAs as biomarkers for PACA.

Expression of LncRNA	Origin	Potential Functions	References
Sox2ot	Peripheral blood	Tumor stage/survival evaluation	[80]
HULC	Peripheral blood	Early diagnosis	[114]
CRNDE	Peripheral blood	Early diagnosis	[117]
MALAT-1	Peripheral blood	Early diagnosis	[117,118]

Even though PACA biomarkers are thought to have potential significance, further research is still needed to confirm how important they are for diagnosing and prognosticating PACA. Ultimately, finding more sensitive and particular exosomal indicators is still a never-ending problem as our understanding of exosomes expands. Even with the significant advancements, there is still much work to be done until the ideal PACA biomarker is found.

## 6. Discussion

Exosomes and lncRNAs play an important role in the development of pancreatic cancer and the mechanism of drug resistance. Exosomes and lncRNAs are involved in tumor angiogenesis, metastasis, and proliferation, and even participate in tumor drug resistance and immune evasion at the genetic level. Although there are many related studies in this field, due to the late start of exosome research, there are still some problems, which can be summarized as the following points: 1. The process of PACA-related exosomal LncRNA cyclization, degradation, and enrichment is not well understood. 2. Utilizing exosomal LncRNA in body fluids as a biomarker for cancer diagnosis and prognosis still has some limitations, such as the fact that there are no standard methods for extracting or detecting exosomes at present. 3. Exosomes may be a double-edged sword for LncRNAs, and we have yet to explore their role.

## 7. Prospect

Some signal pathways associated with lncRNA have been found, but there are still many unknown pathogenesis unelucidated, especially in the early screening, molecular diagnosis, and precision treatment of pancreatic cancer patients. The high content of lncRNAs in the exosomes of pancreatic adenocarcinoma was detected by modern subbiology, biosynthesis, and molecular pharmacology. Molecular, cellular, and dynamical experiments are needed to demonstrate the role of lncRNAs in pancreatic cancer progression in these exosomes and to develop molecular diagnostic tools. Elucidating the role of exosomal lncRNAs in the pathogenesis of pancreatic cancer, discovering new signal pathways and downstream targets, and providing theoretical basis and experimental support for targeted therapy of pancreatic cancer patients [119].

The medical community is increasingly concerned about the gut microbiota, especially as it relates to the function of various organs in the body [120]. Alterations in the microbiota appear to be particularly beneficial for the diagnosis of certain pancreatic pathologies, and future non-invasive diagnostics may emerge from this area. Although not fully understood, the interaction between the microbiota and cancer may involve bacterial metabolites that have a protective effect against tumors [121]. For example, acetate not only alleviates pancreatitis and reduces PDAC risk factors, but also affects epigenetic changes in mesenchymal stem cells that promote their transformation into cancer-associated fibroblasts, thereby increasing the invasiveness of PDAC cells. The gut microbiota can influence cancer through miRNA [122], so the relationship of exosomal lncRNAs between intestinal microecology and cancer may be an interesting scientific issue [123]. It will provide the possibility of preventing and treating cancer by intervening in the intestinal flora and affecting the exosomal lncRNAs.

In addition, the use of new technologies is receiving increasing attention. Delivery of LncRNA to pancreatic adenocarcinoma via exosomes by formulation technology and reduction of production and release of LncRNA from exosomes by gene editing to treat pancreatic adenocarcinoma [124]. It is also a possible future direction.

By revealing the role of exosomal lncRNA in the occurrence, development, treatment, and prognosis of pancreatic adenocarcinoma, as well as the application of new scientific and technological means, it will find new breakthroughs and make greater contributions to the prevention, treatment, and prognosis evaluation of pancreatic cancer.

## 8. Conclusions

In conclusion, exosomal LncRNA has been gradually shown to play a function in pancreatic cancer; it promotes the occurrence and progression of pancreatic cancer in a variety of ways; and its use as a novel therapeutic target and prospective biomarker has also been gradually explored. In the future, the presence of LncRNA in extracellular vesicles will have great potential as a biomarker for cancer diagnosis. The design of targeted therapy for extracellular vesicle drug delivery will also be a hot topic in the future, and its application in immune response, neurological disorders, cardiovascular disease, and other diseases will also be a new frontier in cancer research. This essay examines the function of exosomal LncRNA in pancreatic cancer progression, and it is believed that more and more people will continue to study exosomal LncRNA in the future, which will provide new possibilities in the field of cancer therapy.

## Figures and Tables

**Table 1:** The function of exosomal LncRNAs in PACA.

Exosomal LncRNA	Expression	Function	Target Pathway	References
LncRNA UCA1	Up	Promotes proliferation, migration, invasion and drug resistance	mir-96-5p	[76]
LncRNA SNHG11	Highly expressed	Promotes proliferation, migration, and angiogenesis	miR-324-3p	[77]
LncRNA SBF1-AS2	Down	Inhibit of proliferation	miR-122-5p	[78]
LncRNA LINC00460	Up	Promotes proliferation, migration and invasion	miR-503-5p	[78]
ENST00000560647	Up	Promotes EMT	\	[79]
LncRNA-Sox2ot	Low express	Promotes invasion and metastasis	miR-200 family	[80]
LncRNA-ZFAS1	Up	Promotes metastasis and EMT	\	[81]
LncRNA 01133	Highly expressed	Promotes invasion and EMT	Wnt/β-catenin	[82]
LncROR	up	Promotes gemcitabine resistance	Hippo Signaling	[83]
